# How Do Patients with Chronic Neck Pain Experience the Effects of Qigong and Exercise Therapy? A Qualitative Interview Study

**DOI:** 10.1155/2016/8010891

**Published:** 2016-06-21

**Authors:** Christine Holmberg, Zubin Farahani, Claudia M. Witt

**Affiliations:** ^1^Institute of Public Health, Charité-Universitätsmedizin Berlin, Seestrasse 73, 13347 Berlin, Germany; ^2^Institute for Social Medicine, Epidemiology, and Health Economics, Charité-Universitätsmedizin Berlin, 10098 Berlin, Germany; ^3^Institute for Complementary and Integrative Medicine, University of Zurich and UniversityHospital Zurich, Rämistrasse 100, 8091 Zurich, Switzerland

## Abstract

*Background*. The high prevalence of chronic neck pain in high income countries impacts quality of life and the social and work-related activities of those afflicted. We aimed to understand how mind-body therapies and exercise therapy may influence the experience of pain among patients with chronic neck pain.* Methods*. This qualitative interview study investigated how patients with chronic neck pain experienced the effects of exercise or qigong therapy at two time points: during an intervention at three months and after the intervention at six months. Interviews were analysed thematically across interviews and within person-cases. Based on other qualitative studies, a sample size of 20 participants was deemed appropriate.* Results*. The sample (*n* = 20) consisted of 16 women and four men (age range: 29 to 59). Patients' experiences differed according to the therapies' philosophies. Exercise therapy group interviewees described a focus on correct posture and muscle tension release. Qigong group interviewees discussed calming and relaxing effects. Maintaining regular exercise was easier to achieve with exercise therapy.* Conclusions*. The findings of this study may help health care providers when counselling chronic pain patients on self-help interventions by informing them of different bodily and emotional experiences of mind-body interventions compared to exercise therapy.

## 1. Introduction 

The likelihood of a person in a high income country having unspecific neck pain has been calculated to be on average 49% [1, 2], with women more likely to be affected than men [3, 4]. Chronic neck pain is considered a musculoskeletal disease with biopsychosocial components and a multifactorial aetiology [5, 6]. Older age, being female, high job demands, low social and/or work support, being an ex-smoker, and having a history of lower back disorders and/or neck disorders have all been identified as risk factors for unspecific neck pain [7]. In addition, other studies have found that low socioeconomic status is associated with unspecific neck pain [8, 9]. Severe symptoms of unspecific neck pain include decreased mobility, numbness of limbs, headaches, and migraines [10, 11]. Treatment guidelines for nonspecific neck pain suggest physiotherapeutic manipulation and mobilization [6, 12, 13]. Acupuncture, postisometric relaxation, and muscle building have shown positive effects in the treatment of unspecific neck pain [14]. Similarly, mindfulness exercises have shown mild effects on unspecific neck pain [15].

Since exercise interventions are regularly suggested for the treatment of chronic pain, our research group conducted a randomized controlled trial (RCT) that compared qigong and exercise therapy with a waiting group with respect to improved neck pain, as indicated by the Visual-Analogue Scale (VAS) [16]. In this study, improved neck pain was shown for the qigong group compared to the waiting list group. The exercise group also tended to have an improved VAS compared to the waiting list group. Overall evidence remains scarce, however, and there is still uncertainty about the effects of different forms of exercise on unspecific neck pain [15, 17]. One reason for this is the difficulty in capturing pain experience with one-dimensional pain scales such as the VAS. Such instruments have significant limitations in assessing the complexity of subjective pain experience [18–21]. There is also little information on how exercise interventions may be experienced by patients and how they may influence patients' everyday lives. In clinical research contexts in which complex interventions have been tested [22, 23], study participation has been investigated [20, 24, 25], or if the outcome of the clinical study is difficult to measure based on its subjective nature [26], nested qualitative studies have been added to the RCT research. An important characteristic of qualitative research is its ability to reconstruct individuals' subjective perspectives; it is a methodology suited to capturing individual experiences [27, 28] through narration. Narratives create meaning of events [29]. They are an important means of understanding others' experiences. To elicit the experiences of others in research, qualitative interviewing techniques have been developed using open-ended questions that engage person's experiences [30, 31].

In order to understand in depth how neck pain may be influenced by exercise or qigong therapy, we conducted a qualitative interview study nested within the RCT that looked at the experiences of patients with unspecific chronic neck pain with either qigong or exercise therapy [16], in order to learn about the subjectively experienced effects of both interventions on their pain experience. The experience of pain has been described as all-encompassing, impacting all aspects of a person's life [32, 33]. Given this, how do exercise therapies influence a patient's pain experience?

## 2. Materials and Methods

### 2.1. Study Design

To learn how patients with unspecific chronic neck pain experience and value exercise therapy or qigong, we conducted a nested qualitative study within an RCT [16]. The trial investigated the effect of qigong or exercise therapy compared to a waiting list among patients with chronic neck pain. Patients (*n* = 80) received 18 sessions of either qigong or exercise therapy in a group setting over the course of six months. The assessment of the primary outcome of the RCT was done using the VAS. The qualitative study followed the patients for the duration of the intervention period of the trial.

### 2.2. Data Collection

Semistructured interviews were conducted one-on-one. The interview guideline was developed based on a literature search and on a previously conducted interview study that investigated qigong and exercise therapy in an elderly population with chronic neck pain [20, 34–36]. The interview guideline was tested with four patients and interviews were discussed and evaluated in the research group. The guideline was then revised to ensure that all questions generated answers that reflected subjective experiences. The guideline asked about interviewees' motivation to participate in the trial and their hopes and expectations about trial participation, preexisting experiences with the intervention exercises, changes in pain, exercise behaviour outside of the trial, sleep behaviour, general well-being, concentration, and bodily experiences.

### 2.3. Study Sample

The sample was based on a purposeful sampling strategy. The main variable considered for the selection of interviewees from the trial population was pain intensity as measured by the VAS, the primary outcome of the RCT [16]. An additional selection criterion was gender. The reason for this was that the RCT population consisted of only 12% men (of 122 participants). It seemed important to capture the experience of this underrepresented group in the interviews.

**Figure 1:**
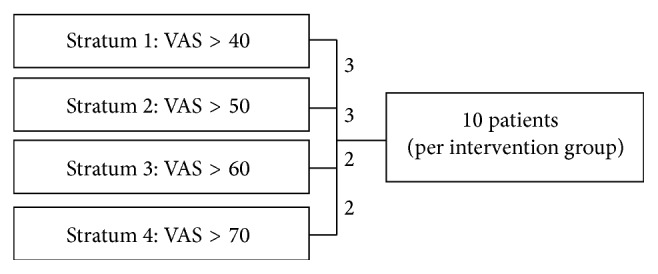
Sampling strategy.

The sample size was determined based on other nested qualitative studies in RCTs, where the sample size ranged between 30 and 40 interviews [37, 38]. To capture experience over time, interviewees were interviewed at two time points. Thus to achieve 40 interviews, 10 interviewees were selected per intervention group resulting in an overall sample size of 20 participants. To identify 20 interview participants, the 80 RCT participants of the intervention groups were sorted into four groups according to their baseline VAS value (Figure 1). To ensure that we had at least one male interviewee per stratum if available, a male person was selected first. The second and third person were selected randomly. We assumed that lower VAS values may reflect pain experiences that are not all-encompassing and not as significant in experiential narratives. For this reason, we chose more interviewees from strata 1 and 2 than from strata 3 and 4.

The ethics committee of the Charité-Universitätsmedizin Berlin approved the study (EA1/2015/07).

### 2.4. Data Collection

Selected interview participants were interviewed after three months and six months of intervention. Interviews were semistructured and qualitative [31, 35]. The guideline was designed to accommodate an interview length of approximately 30 minutes (Table 1). After each interview, the interviewer wrote a protocol summarizing the most important thematic points of the interview as well as context information such as interactions between interviewee and interviewer and emotional involvement. In qualitative research, such protocols are an important means to explore and analyse the relationship between interviewer and interviewee [39]. They are used in analysis to explain and understand the content of the interviews as one way to explicate interviewer influence on the interview situation. Interviews were all conducted at the clinical centre by ZF. For quality purposes, ZF and CH evaluated the interviewing techniques for 25% of the sample. All interviews were audio-recorded and transcribed by the interviewer.

### 2.5. Data Analysis

Data analysis of interviews was conducted inductively based on some of the principles of Grounded Theory such as inductive coding with conceptual codes, category development, and the constant comparison of findings [40–42]. Theoretical and conceptual memos accompanied the coding and analysis process [43].

Data management of transcripts, codes, coding, and memos was done using MAXQDA® Version 10. When new aspects or themes arose in the analysis that led to the development of new codes, all interviews were reread and recoded accordingly. The coding process and category development were regularly discussed in data sessions with a group of qualitative researchers in team meetings to ensure that the analysis was grounded in the data. The importance of conducting data analysis in a group has been stressed as a quality criterion for qualitative research [28]. It is an important tool to explicate different subjective perspectives on qualitative material in order to ensure appropriate data analysis.

## 3. Results

The sample consisted of 16 women and four men, ranging from 29 to 59 years of age, with an average age of 42.6 (±9.8) years. All except one participated in interviews at two time points. The duration of interviewees' neck pain varied between one and five years, with an average of 3.5 (±1.4) years. To reflect the clinical trial sample, 60% of interviewees were selected from the lower VAS strata (40–60 mm) and 40% from the higher strata (60–80 mm).

### 3.1. Experiencing Neck Pain

In all initial interviews, head and neck pain provided the background against which everyday experiences were embedded, and it led to social consequences such as family quarrels due to bad mood, messy rooms, and the inability of interviewees to fulfill their required workload at their jobs.

*I somehow always had these extreme neck pains, and then they expanded to my head, often causing migraines as well. And then I also had these breakdowns making me unable to do anything at all for one or two days (…) I could neither go to work nor concentrate on my studies. So I just lay in bed all day. (29 years old, female)*

*Because it [the pain] always means a loss of income, since you have to be present at work, and that part is of course very uncomfortable as well. (33 years old, male)*

Interviewees described their pain by talking about the difficulties they had with movements, such as turning their head, and sensations in their arms, or talked about occasional headaches resulting from the neck pain. Some interviewees experienced dizziness and impaired vision as well as tinnitus as a result of their pain. In addition, many of the interviewees described sleeping problems.

Overall, eight of the 20 interviewees talked about having severe migraine attacks, which they attributed to their neck pain. Seven of them were in the qigong intervention group and one was in the exercise therapy group.

*Yes, I think that exactly when I have these migraines, then it feels like, it simply grows upwards from the neck and into the head, I really feel it, and I really feel that I (…) am completely strained. Sometimes I think: Is that causing it? (…) That's how it is for me (…) it's connected. (45 years old, female)*

In contrast to the scientific differentiation of pain sites and pain sensations, the interviewees described their pain less in terms of physical symptoms and instead talked about bodily sensations, fear, and feelings of estrangement and betrayal.

*Then I feel like I'm 60 years old (laughs). Sometimes it feels like that. I hate that I have to let it in, because I am really not that old and then I just feel totally inflexible when I am out and about. (30 years old, female)*

Aside from feeling estranged from oneself through the pain, the unpredictability of the pain attacks and not knowing how to handle them impeded social relations and obligations.

*Yes, it has definitely caused me to withdraw (…) and now afterwards, as I have recovered and learned a lot and know much more about how I should handle the discomfort, then it is still, like, I am much more careful and less open to other people, and often say: No, I won't do this to myself, that's too much. So in this regard it has definitely caused me to withdraw in a certain sense. (55 years old, female)*

The experience of helplessness with regard to the pain was a major reason for interviewees to participate in the RCT. Both interventions were something “one could do oneself” and did not involve taking a pill. Working towards actively alleviating pain attacks and being given tools to handle the related suffering were great incentives for participating in the study. This was particularly important because in this sample of interviewees the sudden and uncontrollable onset of pain periods seriously impacted their family life, as well as their work and social relations.

*Yes, because I always wanted to do everything myself. Because I've always been going from doctor to doctor and, and I wanted someone to help me from the outside, and in some way I liked that I could do something on my own – that I didn't always have to be passive. (29 years old, female)*

### 3.2. Intervention Experiences in the Qigong Study Arm

Of the 10 patients in the qigong intervention arm, seven described a difference in their pain experiences after three months of receiving the intervention. They talked about how their everyday life had improved because the pain seemed less dominant. Four of the interviewees attributed a decrease in migraine attacks to the study.

*The acute pains are certainly gone. Just before starting doing qigong, as I went to see the orthopaedist, I could hardly move my neck. It hurt all the way down to the arm, the typical symptoms so to speak, when the pains are there. And that is definitely gone. (52 years old, female)*

Participants mostly described that they experienced a relaxing effect from qigong, both physically and emotionally. Participants experienced physical quietness through the qigong movements, which left them more alert and energized afterwards.

*Yes, afterwards I am much more relaxed. I am not tired anymore. I simply feel that afterwards I have much more energy… In the final phase of my education, I did it more and more frequently, because it felt so good between the different phases of studying, so that was very good. That is how you are supposed to work at university (laughs). (29 years old, female)*

Participants also attributed a perceived change in dealing with stress to qigong and experienced improved and more relaxing sleep. Qigong helped them to maintain a calm attitude and interviewees felt more at ease and balanced. It was this calming effect that some participants associated with an improvement in their strained relationships due to the pain, be it with family or work colleagues.

*But the longer it [doing qigong] lasts, the more pleasant it also gets. Also for your surroundings. I have become much more open, and I am once again compatible with the family. (48 years old, female)*

Since all interviewees talked about the significant impact that their pain experiences had on their lives, it is not surprising that the effects of the intervention, when successful, also included a new sense of joy in life.

*Well, just like before [the pain], you get more life affirming and open and radiate power once again. You know, it was just there again, and it simply returned, slowly and automatically. (48 years old, female)*

Interviewees also described a physical effect of their qigong movements, though this was not considered as important as the abovementioned socioemotional consequences. Overall, participants described having a different feel for their body, in the sense that they had gained an ability to read the signs that their bodies gave them and thus react quickly so that pain attacks would not become as severe as they used to be.

*(…) it suits me perfectly, because I always go there after work. Afterwards, I always feel wonderfully loosened and relaxed, also in my head. Sometimes I would have headaches when I went there, but afterwards they would have dissolved into the air by 8 pm. (30 years old, female)*

In the interviews, participants talked about feeling more in control in general, in particular being able to control their pain more because of their changed bodily experiences. Thus even among patients who did not necessarily feel a decrease in pain overall, their ability to sense a pain attack and know possible self-help interventions was considered positive.

*I still have [pain] like before, but now I can deal with them in a much better way. Because when I feel the discomfort, not just around my neck or in my shoulders, but just when I generally feel weary, then I simply do these qigong exercises and feel how they give me power. And then I feel better. (45 years old, female)*

All of these effects were, however, closely related to doing the exercises. Thus after the intervention terminated, the pain experiences of some of the interviewees worsened compared to during the study. None of the interviewees had kept up a regular practice at home by themselves after study completion. The weekly class had forced them to exercise, and with the lack of such a motivation, other work- or life-related issues became more important than exercising.

*Since I started doing qigong, it [the pain] gradually went away and was almost gone after two months. Which made me very happy. And then it came back in the beginning of September (…) And I have to admit: I haven't been doing my exercises at home regularly (…) I know that I have a tendency to get lazy as soon as the discomfort is gone. (52 years old, female)*

Interestingly, patients were more convinced that qigong had had an effect on their pain experiences in the second interviews. It was the worsening of the symptoms after they stopped the classes that they used as an explanation of why they thought qigong had an effect.

### 3.3. Intervention Experiences in the Exercise Therapy Study Arm

The interviews with those undergoing exercise therapy suggest that the exercises led to a reduction in muscle tensions in the neck, which led to less pain in the region. Similarly, flexibility in the neck region increased. At least six of the patients experienced a reduction in pain during the intervention phase. Headaches were also less severe during this time; one woman even had no headaches during this time. The one interviewee who had migraine attacks continued to have them but experienced them as less severe and bothersome during the intervention phase.

*Well, that's my general impression (…) that it [the pain] is indeed still decreasing, but immediately after having done the exercises I feel that I am able to turn my head much more freely and that my general movements are much more unrestricted. (48 years old, female)*

Interviewees talked about the exercises as being strenuous and some had pain during the workout and muscle aches afterwards. However, these pains were not viewed negatively.

*Sometimes I have met my limits and thought: Damn, why does it hurt so bad when I just raise my arm a bit? Or how can it be this demanding? It is unbelievable (…) But at the same time you realise (…) that now it really hurts, and then my response is, that's just how it is, it has to hurt. I feel the pain and that is just a regular thing. (55 years old, female)*

Similar to the qigong group, the exercises they learned gave interviewees the possibility to act on their pain and do something when pain attacks came. In line with the fact that qigong and exercise therapy are different interventions, both the exercises and how interviewees talked about them also differed, but the positive experience of being able to do something was similar. Contrary to the qigong interviewees, participants in the exercise therapy group talked about the muscle tensions in their neck as a source of their pain and they worked to relax these tensions for a decreased pain experience.

*But it has really done me a lot of good to do these exercises, because I feel that every single muscle gets activated, and I simply get the feeling that: Aha, it is caused by this and this because these specific muscles are shortened or cramped up. And I know that I can do something about it, that I can make it all better, and to me it was all very educational and encouraging. (55 years old, female)*

For some of the interviewees, the feeling of finally not being at the mercy of their pain but having tools to handle it was a relief that influenced their overall well-being and made them more relaxed overall.

*I feel much better and much more comfortable in my body, and I have the feeling that my outward appearance has improved significantly. (48 years old, female)*

The effect of the exercise classes was also present in everyday life, as nine of the interviewees talked about how they were more aware of their body posture and tried to keep an upright back in other situations as well, such as at work or while walking. The exercises helped them to change the way they dealt with their body. Thus the exercises were not a means to rest more but to engage actively and mindfully with their body. All of these changes also led to more restful and peaceful sleep.

*You see, I used to wake up in the middle of the night because my fingers were numb. But now that rarely ever happens. But only when I do the exercises regularly. When I don't do them, then I feel right away that it [the pain] comes back amplified. When I am able to stick to the exercise routine, then everything is simply much better. (59 years old, female)*

In contrast to the qigong intervention, it seemed easier for the exercise therapy group interviewees to integrate the exercises into their daily routine. For example, when running, they would add some of the learned exercises at the beginning or end of their workout. They also did not need extra space to do the exercises, which made it easier to integrate them into their lives.

*And now I have a routine, which consists of doing an exercise here and there, at work, in the sauna or during walks. I simply squeeze an exercise in here and there. (55 years old, female)*

This may be the reason why five of the interviewees in their second interview stated that they continued to experience a positive effect, even with only a biweekly schedule and after the study termination. Others, however, experienced a worsening compared to the first interview. One patient in particular, who had had no pain experience at all during the intervention, had to endure a return of the pain after study termination, albeit less severe than before. Only three of the interviewees talked about continuing their exercises on a regular basis. Thus overall, patients had experienced a positive effect of the intervention, which continued if they continued exercising. However, this was very difficult for many in the group due to different priority setting after study termination and other illnesses or simply because they missed the class and the peers with whom the exercise was more fun.

## 4. Discussion

In this sample of patients who had experienced chronic neck pain for a long period of time, an intervention with either qigong or exercise therapy was described as being beneficial for personal well-being. In particular, interviewees talked about having gained a sense of how they may be able to improve their pain experience themselves through the learned exercises. In both groups, the effects of the intervention influenced body sensations and the ways in which interviewees dealt with their bodies in everyday life. In line with the respective interventions, the changed ways of dealing with pain differed by intervention. The exercise therapy group was overall more aware of their body posture and worked towards reducing muscle tension. This is in line with the more mechanistic view of the body in exercise therapy. The qigong intervention group interviewees discussed being calm and balanced. Again, this fits well with the philosophical background of qigong, which is embedded within a holistic view of Chinese medicine. Contrary to the exercise therapy group, the interviewees from the qigong intervention arm talked about a noticeable improvement in their social relations and social and work situations. For relief and improvements in everyday life, such as improved sleeping patterns, both interventions seemed to work for the interviewee groups. Similarly, however, in both interview groups participants found it difficult to maintain their exercise routine after the weekly classes came to an end. During the period of the classes, interviewees in the exercise therapy intervention arm found it easier to exercise outside of class, but this was not sustained for most after the intervention was complete. A group setting with a regular schedule, if possible weekly, provided the stability and ability for participants in both groups to prioritize the exercises, which seemed necessary to show an improvement in interviewees' everyday lives.

In both groups, the experience of pain impacted their everyday lives, so it is not surprising that the effects of the interventions were not only of pain reduction but much more about a feeling of improved well-being overall. Interviewees also explained the feeling of well-being in terms of the fact that they had learned tools to handle pain attacks and reduce their impact, which in turn gave them a feeling of empowerment rather than that of being a victim of pain. Feeling in control and being active in their endeavour to deal with their pain were an important side effect of the intervention.

Considering the high prevalence of chronic pain and its impact on quality of life, including the impairments it implies in terms of work and social life, interventions that can be learned and used easily are important. The current interviews provide a nuanced description of how different exercises may influence everyday experiences in subtly different ways.

Chronic pain is influenced by somatic, behavioural, and social factors [44]. Thus pain therapy should focus not only on reducing pain but also on providing tools to influence behavioural and social factors, such as coping with stress, that patients may use themselves [12, 45]. The RCT of our study group had shown that both interventions have a positive effect on pain intensity [16]. In addition, the interviews now provide insights into how this difference in pain intensity was experienced in everyday life. In particular, the interviews showed that the learned exercise tools helped the interviewees to feel empowered by having simple tools to deal with their pain attacks. For the qigong intervention group, patients experienced a difference in their everyday lives due to feeling calmer and more balanced. A systematic review of mind-body interventions such as qigong and tai chi has also described such a positive impact on psychological well-being [46]. On the other hand, such exercises were more difficult for patients to keep up after the intervention was completed, as they necessitated a special framework in which to do them. In contrast, interviewees in the exercise therapy intervention arm were more able to maintain an exercise routine outside of class. Considering that interviewees in both intervention groups described the necessity of regular exercise in order to maintain the positive effects they experienced, the ability to integrate the exercises into a regular schedule may be of importance.

This qualitative study aimed to understand how qigong and exercise therapy influence the experience of pain, selecting 20 persons from an RCT population. Since the RCT population consisted of mainly female participants, this is also true for the qualitative sample. It is very likely that if more men could have been included in this study, other experiences would have been reported. In this study the interviews of the four men did not differ in how they spoke about their experiences in this study compared to the other interviewees. Similarly, it was a male physician who conducted the interviews; this is likely to have impacted what was narrated and how. A different interviewer would have likely received other nuances of the patients' experiences. To understand this influence, the interviewer wrote a context protocol for each interview. Furthermore, to improve data collection, ZF, CW, and CH held team meetings in which they listened to the interviews. Finally, the purposeful sampling strategy led to the selection of particular individuals from the RCT population. It is possible that selection based on other characteristics such as age would have led to other study results. It is also possible that a larger sample size could have increased the experienced effects of both interventions. Another study of our group that investigated how qigong and exercise therapy influence pain experience in the elderly found similar results to the ones described here, which does suggest that such different experiences by exercise intervention may be found across different study populations [36].

## 5. Conclusions

There is a need for tools that pain patients can use themselves in order to improve their pain and alleviate some of the negative aspects of the pain in their everyday lives. Physical activity, in particular mind-body interventions and exercise therapy, has been shown in other studies to be an effective tool for pain patients. However, thus far little is known about how the reduction in pain intensity that has been shown in RCTs may be experienced by individuals and if there are different experiences depending on what type of exercise is used for pain alleviation.

The presented qualitative study shows that interviewees discussed the effects of the exercises on their pain experience in everyday life in line with the philosophical or theoretical underpinnings of the different interventions. Both exercise forms may give patients a feeling of empowerment because they have tools that they can use themselves to alleviate their pain. While qigong seemed to have an overall calming effect on interviewees, exercise therapy was easier to maintain after the intervention ended. Such aspects may be of importance when health care providers discuss different options with their patients to alleviate pain. To better understand these subtle differences in pain experiences, additional studies are necessary among other study populations, including male pain patients.

## Figures and Tables

**Table 1:** Comparison of the interview group with the study sample [16].

	Qigong	Exercise therapy	Study population
	Mean (±SD)	Mean (±SD)	Mean (±SD)
	*N* = 10 8 female, 2 male	*N* = 10 8 female, 2 male	*N* = 122 107 female, 25 male
Age (years)	40.6 (±10.3)	44.6 (±9.3)	45.6 (±10.7)
VAS baseline (mm)	60.1 (±13.1)	56.5 (±12.2)	56.2 (±14.1)
Pain duration (years)	3.9 (±1.2)	3.1 (±1.6)	3.2 (±1.6)

## References

[1] Picavet H. S. J., Schouten J. S. A. G. (2003). Musculoskeletal pain in the Netherlands: prevalences, consequences and risk groups, the DMC3-study. *Pain*.

[2] Borghouts J. A. J., Koes B. W., Vondeling H., Bouter L. M. (1999). Cost-of-illness of neck pain in The Netherlands in 1996. *Pain*.

[3] Fejer R., Kyvik K. O., Hartvigsen J. (2006). The prevalence of neck pain in the world population: a systematic critical review of the literature. *European Spine Journal*.

[4] Jensen I., Harms-Ringdahl K. (2007). Strategies for prevention and management of musculoskeletal conditions. Neck pain. *Best Practice & Research Clinical Rheumatology*.

[5] Robert Koch-Institut (2002). Chronische schmerzen—kopf- und rückenschmerzen, tumorschmerzen. *Gesundheitsberichterstattung des Bundes*.

[6] Pool J. J. M., Rubinstein S. M., van Tulder M. (2005). Anerkannte Evidenz der Wirksamkeit konservativer Behandlungen akuter und chronischer Nackenschmerzen. *Manuelle Medizin*.

[7] McLean S. M., May S., Klaber-Moffett J., Sharp D. M., Gardiner E. (2010). Risk factors for the onset of non-specific neck pain: a systematic review. *Journal of Epidemiology and Community Health*.

[8] Siivola S. M., Levoska S., Latvala K., Hoskio E., Vanharanta H., Keinänen-Kiukaanniemi S. (2004). Predictive factors for neck and shoulder pain: a longitudinal study in young adults. *Spine*.

[9] Croft P. R., Lewis M., Papageorgiou A. C. (2001). Risk factors for neck pain: a longitudinal study in the general population. *Pain*.

[10] Wells G. A., Tugwell P., Brosseau L. (2001). Philadelphia panel evidence-based clinical practice guidelines on selected rehabilitation interventions: overview and methodology. *Physical Therapy*.

[11] Agosti R. (2000). Zervikales Kopfweh—science oder fiction?. *Schweizerische Ärztezeitung*.

[12] Seeger D., Pfingsten M., Mann K., Hildebrandt J. (2003). Behandlung von chronischen HWS-Beschwerden. *Manuelle Medizin*.

[13] Ylinen J. (2007). Physical exercises and functional rehabilitation for the management of chronic neck pain. *Europa Medicophysica*.

[14] Witt C. M., Jena S., Brinkhaus B., Liecker B., Wegscheider K., Willich S. N. (2006). Acupuncture for patients with chronic neck pain. *Pain*.

[15] Gross A., Kay T. M., Paquin J. P. (2015). Exercises for mechanical neck disorders. *The Cochrane Database of Systematic Reviews*.

[16] Rendant D., Pach D., Lüdtke R. (2011). Qigong versus exercise versus no therapy for patients with chronic neck pain: a randomized controlled trial. *Spine*.

[17] Hurwitz E. L., Carragee E. J., van der Velde G. (2009). Treatment of neck pain: noninvasive interventions. Results of the Bone and Joint Decade 2000–2010 Task Force on Neck Pain and Its Associated Disorders. *Journal of Manipulative and Physiological Therapeutics*.

[18] Williams A. C. D. C., Davies H. T. O., Chadury Y. (2000). Simple pain rating scales hide complex idiosyncratic meanings. *Pain*.

[19] Robinson-Papp J., George M. C., Dorfman D., Simpson D. M. (2015). Barriers to chronic pain measurement: a qualitative study of patient perspectives. *Pain Medicine*.

[20] Holmberg C., Karner J. J., Rappenecker J., Witt C. M. (2014). Clinical trial participants' experiences of completing questionnaires: a qualitative study. *BMJ Open*.

[21] Dijkers M. (2010). Comparing quantification of pain severity by verbal rating and numeric rating scales. *The Journal of Spinal Cord Medicine*.

[22] Nelson G., Macnaughton E., Goering P. (2015). What qualitative research can contribute to a randomized controlled trial of a complex community intervention. *Contemporary Clinical Trials*.

[23] Murtagh M. J., Thomson R. G., May C. R. (2007). Qualitative methods in a randomised controlled trial: the role of an integrated qualitative process evaluation in providing evidence to discontinue the intervention in one arm of a trial of a decision support tool. *Quality & Safety in Health Care*.

[24] Guillemin M., Barnard E., Walker H., Bennell K., Hinman R., Gillam L. (2015). Participants' understanding of informed consent in a randomized controlled trial for chronic knee pain. *Journal of Empirical Research on Human Research Ethics*.

[25] Holmberg C., Whitehouse K., Daly M., Mccaskill-Stevens W. (2015). Gaining control over breast cancer risk: transforming vulnerability, uncertainty, and the future through clinical trial participation - a qualitative study. *Sociology of Health and Illness*.

[26] Midgley N., Ansaldo F., Target M. (2014). The meaningful assessment of therapy outcomes: incorporating a qualitative study into a randomized controlled trial evaluating the treatment of adolescent depression. *Psychotherapy*.

[27] Meyer T., Karbach U., Holmberg C. (2012). Qualitative research in health services research—discussion paper, part 1: what is the idea?. *Gesundheitswesen*.

[28] Stamer M., Guthlin C., Holmberg C., Karbach U., Patzelt C., Meyer T. (2015). Qualitative research in health services research—discussion paper, part 3: quality of qualitative research. *Gesundheitswesen*.

[29] Greenhalgh T., Hurwitz B. (1999). Narrative based medicine: why study narrative?. *British Medical Journal*.

[30] Green J., Thorogood N. (2009). *Qualitative Methods for Health Research*.

[31] Hopf C., Flick U. (2007). Qualitative Interviews—ein Überblick. *Qualitative Forschung ein Handbuch*.

[32] Delvecchio M. J., Good P., Brodwin P., Good B., Kleinman A., Delvecchio M. J., Good P., Brodwin P., Good B., Kleinman A. (1992). Pain as human experience: an introduction. *Pain as Human Experience An Anthropological Perspective*.

[33] Good B. J. (1995). *Medicine, Rationality and Experience: An Anthropological Perspective*.

[34] Flick U., von Kardorff E., Steinke I. (2007). *Qualitative Forschung. Ein Handbuch*.

[35] Kvale S. (1996). *Interviews-An Introduction to Qualitative Research Interviewing*.

[36] Holmberg C., Rappenecker J., Karner J. J., Witt C. M. (2014). The perspectives of older women with chronic neck pain on perceived effects of qigong and exercise therapy on aging: a qualitative interview study. *Clinical Interventions in Aging*.

[37] Hughes J. G., Russell W., Breckons M., Richardson J., Lloyd-Williams M., Molassiotis A. (2014). ‘I assumed that one was a placebo’: exploring the consent process in a sham controlled acupressure trial. *Complementary Therapies in Medicine*.

[38] Rise M. B., Evensen G. H., Moljord I. E. O., Rø M., Bjørgen D., Eriksen L. (2014). How do patients with severe mental diagnosis cope in everyday life—a qualitative study comparing patients' experiences of self-referral inpatient treatment with treatment as usual?. *BMC Health Services Research*.

[39] Miles M. B., Huberman A. M. (1994). *Qualitative Data Analysis: An Expanded Sourcebook*.

[40] Glaser B. G., Strauss A. L. (2010). *Grounded Theory. Strategien qualitativer Forschung*.

[41] Böhm A., Flick U. (2007). Theroetisches codieren: textanalyse in der grounded theory. *Qualitative Forschung. Ein Handbuch*.

[42] Lamnek S. (2005). *Qualitative Sozialforschung. Lehrbuch*.

[43] Strübing J. (2008). Was ist grounded theory?. *Grounded Theory: Zur Sozialtheoretischen und Epistemiologischen Fundierung des Verfahrens der Empirisch Begründeten Theoriebildung*.

[44] Seidenspinner D. (2005). Psychologische Aspekte. *Training in der Physiotherapie*.

[45] Rogers C. E., Larkey L. K., Keller C. (2009). A review of clinical trials of tai chi and qigong in older adults. *Western Journal of Nursing Research*.

[46] Stenlund T., Birgander L. S., Lindahl B., Nilsson L., Ahlgren C. (2009). Effects of Qigong in patients with burnout: a randomized controlled trial. *Journal of Rehabilitation Medicine*.

